# Acceptance of COVID-19 vaccine among sub-Saharan Africans (SSA): a comparative study of residents and diasporan dwellers

**DOI:** 10.1186/s12889-023-15116-w

**Published:** 2023-01-28

**Authors:** Chundung Asabe Miner, Chikasirimobi G. Timothy, Khathutshelo Percy, Mashige, Uchechukwu Levi Osuagwu, Esther Awazzi Envuladu, Onyekachukwu Mary-Anne Amiebenomo, Godwin Ovenseri-Ogbomo, Deborah Donald Charwe, Piwuna Christopher Goson, Bernadine N. Ekpenyong, Emmanuel Kwasi Abu, Raymond Langsi, Richard Oloruntoba, Tanko Ishaya, Kingsley E. Agho

**Affiliations:** 1 Department of Community Medicine, College of Health Sciences, University of Jos, Jos, Plateau State Nigeria; 2 Department of Optometry, Mzuzu University, Mzuzu, Malawi; 3 African Vision Research Institute (AVRI), School of Health Sciences, University of KwaZulu-Natal, Durban, South Africa; 4 Bathurst Rural Clinical School, School of Medicine, Western Sydney University, Bathurst, NSW 2795 Australia; 5 Translational Health Research Institute (THRI), Western Sydney University, 2506 New South Wales, Australia; 6 Department of Community Medicine, College of Health Sciences, University of Jos, Jos, Nigeria; 7 School of Optometry and Vision Sciences, College of Biomedical Sciences, Cardiff University, Cardiff, UK; 8 Department of Optometry, Faculty of Life Sciences, University of Benin, Benin City, Ugbowo Nigeria; 9 Department of Optometry, Centre for Health Sciences, University of the Highlands and Islands, Inverness, UK; 10 Tanzania Food and Nutrition Center, Dar-Es-Salaam, Tanzania; 11 Department of Psychiatry, College of Health Sciences, University of Jos, Jos, Plateau State Nigeria; 12 Department of Public Health, Faculty of Allied Medical Sciences, College of Medical Sciences, University of Calabar, Cross River State, Calabar, Nigeria; 13 Department of Optometry and Vision Science, School of Allied Health Sciences, College of Health and Allied Sciences, University of Cape Coast, Cape Coast, Ghana; 14 Health Division, University of Bamenda, Bambili, Cameroon; 15 School of Management and Marketing, Curtin University, Kent Street, Bentley, WA 6102 Australia; 16 Department of Computer Science, University of Jos, Jos, Nigeria; 17 School of Health Science, Western Sydney University, Campbelltown, Australia

**Keywords:** Vaccination, Acceptance, COVID-19, Hesitancy, Resistance, Sub-Sahara Africa, Locals, Diaspora

## Abstract

**Background:**

The COVID-19 vaccines are being rolled out across all the sub-Saharan Africa (SSA) countries, with countries setting targets for achieving full vaccination rates. The aim of this study was to compare the uptake of, resistance and hesitancy to the COVID-19 vaccine between SSA locally residents and in the diasporan dwellers.

**Methods:**

This was a cross-sectional study conducted using a web and paper-based questionnaire to obtain relevant information on COVID-19 vaccine acceptance. The survey items included questions on demography, uptake and planned acceptance or non-acceptance of the COVID-19 vaccines among SSAs. Multinomial logistic regression was used to determine probabilities of outcomes for factors associated with COVID-19 vaccination resistance and hesitancy among SSA respondents residing within and outside Africa.

**Results:**

Uptake of COVID-19 vaccines varied among the local (14.2%) and diasporan (25.3%) dwellers. There were more locals (68.1%) who were resistant to COVID-19 vaccine. Participants’ sex [adjusted relative risk (ARR) = 0.73, 95% CI: 0.58 – 0.93], education [primary/less: ARR = 0.22, CI:0.12 – 0.40, and bachelor’s degree: ARR = 0.58, CI: 0.43 – 0.77]), occupation [ARR = 0.32, CI: 0.25—0.40] and working status [ARR = 1.40, CI: 1.06—1.84] were associated with COVID-19 vaccine resistance among locals. Similar proportion of local and diasporan dwellers (~ 18% each) were hesitant to COVID-19 vaccine, and this was higher among health care workers [ARR = 0.25, CI: 0.10 – 0.62 and ARR = 0.24, CI:0.18—0.32, diaspora and locals respectively]. After adjusting for the potential confounders, local residents aged 29–38 years [ARR = 1.89, CI: 1.26—2.84] and lived in East Africa [ARR = 4.64, CI: 1.84—11.70] were more likely to report vaccine hesitancy. Knowledge of COVID vaccines was associated with hesitancy among local and diasporan dwellers, but perception was associated with vaccine resistance [ARR = 0.86,CI: 0.82 – 0.90] and hesitancy [ARR = 0.85, CI: 0.80 – 0.90], only among the local residents.

**Conclusions:**

Differences exist in the factors that influence COVID-19 vaccine acceptance between local SSA residents and thediasporan dwellers. Knowledge about COVID-19 vaccines affects the uptake, resistance, and hesitancy to the COVID-19 vaccine. Information campaigns focusing on the efficacy and safety of vaccines could lead to improved acceptance of COVID-19 vaccines.

**Supplementary Information:**

The online version contains supplementary material available at 10.1186/s12889-023-15116-w.

## Introduction

The coronavirus disease (COVID-19) pandemic that started in December of 2019, initially reported in Wuhan, China, has continued despite preventative measures adopted worldwide under the guidance of the World Health Organization (WHO). Many countries have experienced their second, third and fourth waves in terms of cases and resultant deaths [1–4]. The outbreak of the new Omicron variant in different countries [5–7] is of global concern [8], as it threatens the return to normalcy and the ongoing COVID-19 vaccination programmes. Non-pharmaceutical interventions to minimise the spread of infections included travel restrictions, lockdowns, physical distancing, regular handwashing and wearing of face masks [9, 10]. From the onset of the pandemic, scientists and pharmaceutical companies began the development of COVID-19 vaccines to offer protection against severe disease [11] .

The Pfizer/BioNTech, Moderna, AstraZeneca/Oxford, Johnson &Johnson, Sinopharm/BIBP and India’s Covishield [12, 13], vaccines are licensed for use across the globe. The utilisation of any vaccine can be influenced by system, client and provider factors [14], but in particular, vaccine acceptance plays a huge role for clients and providers. Generally, the acceptance of any vaccine has been shown to be influenced by demographic factors, knowledge of the disease and the consequences of contracting it, perceptions of susceptibility, potential benefits of a health action and the occurrence of one or more cues to action [11–17]. Similar factors may influence COVID-19 vaccine acceptance.

The COVID-19 vaccines have been shown to be efficient and safe [18], however, their acceptance is a major barrier to the successful rollout plans in different countries including the SSA region. This is further exacerbated by the mistrust in governments demonstrated by residents in this sub-region [19]. The WHO defines vaccine hesitancy as a ‘delay in acceptance or refusal of safe vaccines despite availability of vaccine services’[20] It is also stated to be one of the top ten threats to global health [21, 22]. Vaccine hesitancy is used to describe a phenomenon where individuals are unsure of getting vaccinated. Those who object to getting the vaccine are defined as vaccine resistant [23].

The success of vaccines depends on achieving maximum coverage and thereby attaining herd immunity [24]. Vaccine acceptance is therefore crucial to the efforts currently being made by public health experts of ensuring that the communities in every country are fully vaccinated. Studies have shown that there have been disparities in vaccine acceptance for other conditions, and factors such as age, race and ethnicity, social class, country and region of origin were associated with acceptance of vaccines [25, 26]. Similar results were reported for COVID-19 vaccines [27, 28].

Persons in the diaspora are “national migrant communities living in interaction among themselves and with their country of origin” [29]. Africans in the diaspora have been referred to by the African Union as “people of African origin living outside of the continent, irrespective of their citizenship and nationality, and who are willing to contribute to the development of the continent and the building of the African Union” [30]. It is generally believed that being in the diaspora provides Africans with greater opportunities to become more enlightened and therefore adopt different approaches to decision making [30]. Furthermore, studies have shown that there is geographical and spatial variation in the uptake of vaccines [31, 32]. In SSA, access to COVID-19 vaccines have improved, but the availability of vaccines and uptake remains substantially low compared with the rich European and North-American countries [33], and only 11% of the adult population in Africa were fully vaccinated as at January 2021 [34]. Although there are significant differences in the vaccination programmes and their rollout between countries [35, 36], the fact that a previous study found similarities in the attitude and risk perception towards COVID-19 among Africans living locally and those in the diaspora (mostly living in Western countries) during the lockdown, [37] suggests there could be similarities in their acceptance of the COVID-19 vaccination.

In low-middle income countries such as Nigeria which is in SSA, and others like India, Bangladesh (76.7%) and Egypt (42.6%), varying vaccine acceptance rates have been reported. Risks of COVID-19 infection and being male were factors that influenced COVID-19 vaccine acceptance (Patwary et al., 2022). Willingness to accept the COVID-19 vaccine was found to be high in Low-Middle income countries compared to USA and Russiaas reported by Solis Arce et al., 2022. Behaviour changes and outlooks in combination with perceived risks of COVID-19 disease informed the decision to accept the COVID-19 vaccine or not in most low-middle income countries of which most SSA countries are classified (Patwary et al., 2021). All those studies focused on the indigene and did not compare how the locals in a setting and indigenes living in locations outside their birth places perceive COVID-19 vaccination. This study, therefore, sought to investigate the differences in the acceptance of COVID-19 vaccination of sub-Sahara Africans living on the African continent and the diasporan dwellers. Although different studies exist that looked at COVID-19 vaccine acceptance, none had compared the same between SSAs who are resident locally and the diasporan dwellers at the time of this study.

## Methods

### Design and setting of the study

This was a web-based and paper-based cross-sectional survey carried out between 14^th^ of March and 17^th^ of May 2021. Due to the continued COVID-19 lockdowns in many of the target countries at the time of this study, web-based study was most appropriate even though it may have excluded some participants with no access to internet-based phone/computer services.

### Characteristics of participants

The study population included adults who were 18 years and older, and living in sub-Saharan Africa countries (local residents) and those living in the diaspora (outside of Africa). Respondents from several countries in SSA, mostly from Cameroun, Ghana, Nigeria, South Africa, Tanzania, and those in diaspora mostly living in Australia, United Kingdom, United States, Saudi Arabia, Canada, China, and India took part in this study.

### Sample size determination

The sample size was determined using Cochran's formulae (*n* = z^2^pq/d^2^) with the assumption of a proportion of 50% at a confidence level of 95% with an error margin of 2.5%. A 20% non-response rate was assumed, and a minimum sample size of 2401 was obtained.

### Survey instrument and data collection

Data was collected using a validated self-administered questionnaire adapted from a previous study [38]. The survey tool was tested for the internal validity of the items, and Cronbach's alpha coefficient score ranged from 0.70 and 0.74, indicating satisfactory consistency [39]. The questionnaire was designed on survey monkey in both English and French, which are spoken languages in 26 and 21 SSA countries, respectively [40]. The questionnaire was disseminated electronically through an e-link on social media networks such as WhatsApp, Facebook and e-mail. There was an accompanying introductory section that included the background and goal of the study, procedure for participation and informed consent guide. Participants were requested on the introductory page not to participate in the survey more than once.

### Confounding variables

The survey instrument showing the various variables collected has been presented as a supplementary material (Supplementary Table 1 [S1 Table]). The independent variables included sociodemographic variables; age, gender, region, marital status, the highest level of education, occupation, employment status, religion, smoking status, previous vaccination for other conditions and pre-existing medical conditions; knowledge of COVID-19 vaccines; perception of risk for contracting COVID-19; and attitude towards vaccination for COVID-19. Details of these variables were described in S1 Table. The exposure variable was the ‘place of residence’ (local or diaspora).

The COVID-19 vaccine knowledge items had 10 questions on a Likert scale with five levels as indicated in SI Table 1. The scores for nine of the items ranged from 0 (lowest) to 4 (highest) while, for one item, it was coded as 1 for Yes and 0 for No. The overall knowledge towards COVID-19 vaccination score ranged from 0 -37 points, with a higher knowledge score indicating a better knowledge towards COVID-19 vaccination.

The attitude towards the COVID-19 vaccine items included four items with each assigned 2 points for ‘yes’, 1 point for ‘unsure’ and 0 point for ‘No’. The total attitude score ranged from 0 to 8, with a higher score denoting a better attitude towards COVID-19 vaccination.

The risk perception for contracting the disease after vaccination included questions on how the participants rate their risk of becoming infected with the virus and risk of dying from the infection. The responses were structured using a Likert scale with five levels (S1 Table), with scores for each item ranging from 0 (lowest) to 4 (highest). The total perception score ranged from 0 to 8, with a higher score representing a higher perception of contracting the infection following COVID-19 vaccination.

### Main outcome variables

The main outcome variables were vaccine uptake, resistance and hesitancy. Uptake was determined by answering ‘yes’ to the question “Have you been vaccinated against COVID-19?”. The vaccine resistant group were those that answered ‘no’ to the question ‘Will you be willing to be vaccinated against COVID-19 if the vaccine becomes available in your country?’, while those who answered ‘not sure’ were defined as the vaccine ‘hesitant’ group.

### Statistical analysis

Data were analyzed using STATA/MP version 14 (Stata Corp 2015, College Station, TX, USA). A 95% confidence interval (CI) was set for this survey, and a *P*-value of < 0.05 was considered statistically significant. Descriptive data were summarized and presented in tables and charts using frequencies, percentages, mean and standard deviations as required. Multinomial logistic regression analyses were used to examine the COVID-19 vaccination status on sources of information. As part of the multiple multinomial logistic regression analyses, a staged modelling technique was carried out. Elimination method was conducted using multiple multinomial logistic regression modelling techniques to remove statistically non-significant variables. Demographic factors were first entered into the baseline multiple regression model, followed by health indicator factors and the exposure variables were examined in the final model, which also included knowledge, attitude and risk perception variables, keeping only those variables significant in the previous model. In the final model, we tested and reported any co-linearity. The relative risk with 95% confidence intervals were calculated to assess the adjusted risks of independent variables.

## Results

### Characteristics of the respondents

There were a total of 2545 SSA respondents [2391 locals (93.9%) and 154 in the diaspora (6.1%)]. Table 1 shows the frequency and percentage distribution of respondents according to their socio-demographic variables. The majority of the SSA local residents (67.8%) were younger than 38 years, while those in the diaspora were older. There were more females than males in both groups, and the majority were originally from West Africa (locals 55.6%, diaspora 75.4%). More than half (56.9%) of the locals were not married, and 59.7% from the diaspora were married. Many locals had a bachelor’s degree (56.9%), and most diasporan participants were postgraduate degree holders (53.3%). Most respondents from both groups were employed /self-employed, predominantly non-healthcare workers and of the Christian faith. More than 80% of locals and above 90% of those in the diaspora had been previously vaccinated for one or two other conditions. More than two-thirds of the respondents indicated that they have never smoked. A higher proportion of respondents with preexisting conditions were locally resident (84.6%) compared to those in the diaspora (69.5%). *Chi-square* test results shown in Table 1 revealed statistically significant differences between the local and diasporan dwellers across all sociodemographic variables (*P* < 0.05, for all comparisons), with the exception of religion (*P* = 0.966).

**Table 1:** Characteristics (*n* = 2545) of the study participants living in (Local) and outside of Africa (Diaspora)

Variables	Local 2391 (93.9%)	Diaspora 154 (6.1)	*P*-value^^^
**Age group (years)**			
18 – 28	898 (38.7)	23 (14.9)	< 0.001
29 – 38	677 (29.1)	41 (26.6)	
39 – 48	450 (19.4)	46 (29.9)	
49 +	297 (12.8)	44 (28.6)	
**Sex**			
Males	1,264 (52.9)	112 (72.7)	< 0.001
Females	1,127 (47.1)	42 (27.7)	
SSA region of origin^*^			
West Africa	1,330 (55.6)	107 (75.4)	< 0.001
East Africa	116 (4.9)	6 (4.2)	
Central Africa	288 (12.1)	24 (16.9)	
Southern Africa	657 (27.5)	5 (3.5)	
**Marital status**			
Married	1,030 (43.1)	92 (59.7)	< 0.001
Not married ǂ	1,361 (56.9)	62 (40.3)	
**Highest level of education**			
Postgraduate degree (Masters/PhD)	668 (27.9)	82 (53.3)	< 0.001
Bachelor’s degree	1,237 (51.7)	61 (39.6)	
Secondary/High School	436 (18.2)	9 (5.8)	
Primary or Less	50 (2.1)	2 (1.3)	
**Employment status**			
Employed/Self-employed	1,733 (72.5)	139 (90.3)	< 0.001
Unemployed/Retired	658 (27.5)	15 (9.7)	
**Religion**			
Christianity	2,140 (89.5)	138 (89.6)	0.966
Others	251 (10.5)	16 (10.4)	
**Occupation**			
Non-Healthcare	1,658 (69.3)	95 (61.7)	0.047
Healthcare	733 (30.7)	59 (38.3)	
**Previous vaccination for any condition**			
No	430 (18.0)	15 (9.7)	0.009
Yes	1,961 (82.0)	139 (90.3)	
**Smoking Status**			
Ex-smoker	142 (5.9)	18 (11.7)	0.014
Current smoker	168 (7.0)	8(5.2)	
Non-smoker	2,081 (87.0)	128 (83.1)	
**Risk factors: Any pre-existing condition**^**§**^			
No	2,022 (84.6)	107 (69.5)	< 0.001
Yes	369 (15.4)	47 (30.5)	

Data presented as frequencies (percentages)

^^^chi-square test were used to obtained the *P*−value

^*^SSA Sub−Sahara Africa

^ǂ^includes single, divorced, and widowed

^**§**^includes the presence of any of the following conditions: cancer, diabetes, hypertension, asthma, kidney disease, any heart condition, sickle cell anemia

### Prevalence of uptake, resistance and hesitancy towards COVID-19 vaccine in SSA

**Fig. 1:**
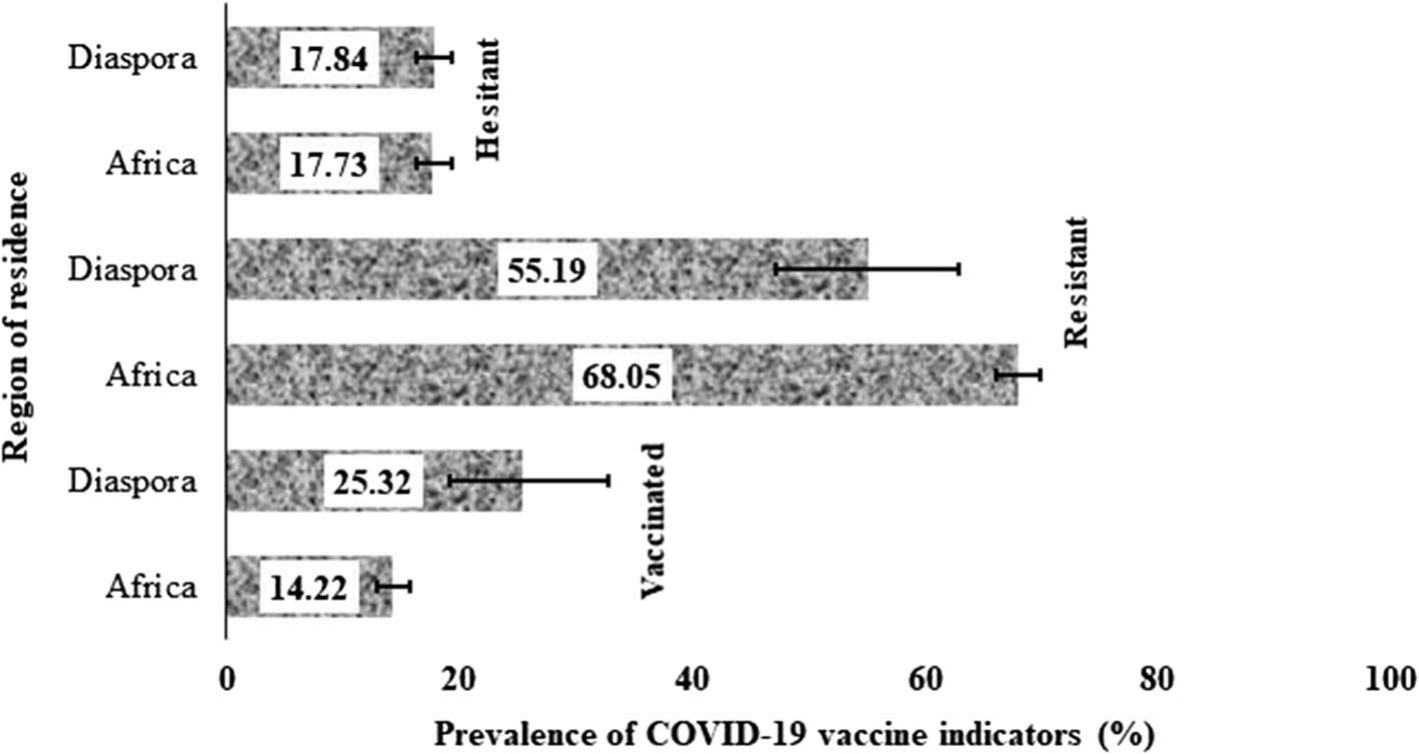
Prevalence and 95% confidence intervals of vaccine uptake, resistance and hesitancy among SSAs living in (within) and outside of Africa (diaspora)

Figure 1 presents the prevalence of vaccine uptake, resistance and hesitancy in both locals and those in the diaspora. The prevalence of COVID-19 vaccine uptake was almost twice higher among the diasporans (25.3%) than among the locals (14.2%). Resistance to the COVID-19 vaccine was more common among the locals (68.1%) than those in the diaspora (55.2%). Hesitancy to COVID-19 vaccine was almost the same for both locals and resident in the diaspora (See Fig. 1).

### Distribution of vaccine uptake, resistance and hesitancy among local and diasporan residents

Table 2 shows the variations in the distribution of vaccine uptake, resistance and hesitancy across the demographic variables as well as their mean scores for knowledge, attitude and perception of risk of infection. Those aged between 39 – 48 years had the highest proportion of locals that were resistant to the vaccine (70.0%) while among those in the diaspora, the 18 – 28 years’ age range had the highest proportion (73.9%). More males (70.2%) than females (65.7%) were resistant to taking the vaccine among the locals, whereas there was a preponderance of resistant females in the diasporan group (64.3%).

**Table 2:** Prevalence of vaccine uptake, hesitancy and resistance among SSAs living in (local) and outside Africa (diaspora)

**Variable**		**LOCAL**			**DIASPORA**	
	**Uptake** *n* = 340	**Resistant** *n* = 1627	**Hesitant** *n* = 424	**Uptake** *n* = 39	**Resistant** *n* = 85	**Hesitant** *n* = 30
**Age in years**						
**18 – 28**	127 (14.1)	621 (69.2)	150 (16.7)	2 (8.7)	17 (73.9)	4 (17.0)
**29 – 38**	101 (14.9)	435 (64.3)	141 (20.8)	3 (7.3)	25 (61.0)	13 (31.0)
**39 – 48**	61 (13.6)	315 (70.0)	74 (16.4)	18 (39.1)	20 (43.5)	8 (17.0)
**49 + **	33 (11.1)	205 (69.0)	59 (19.9)	16 (36.4)	23 (52.3)	5 (11.0)
**Sex**						
**Males**	159 (12.6)	887 (70.2)	218 (17.3)	32 (28.6)	58 (51.8)	22 (19.6)
**Females**	181 (16.1)	740 (65.7)	206 (18.3)	7 (16.7)	27 (64.3)	8 (19.1)
**Region**						
**West Africa**	205 (15.4)	897 (67.4)	228 (17.1)	32 (29.9)	54 (50.5)	21 (19.6)
**East Africa**	6 (5.2)	80 (69.0)	30 (25.9)	0 (0.0)	3 (50.0)	3 (50.0)
**Central Africa**	58 (20.1)	186 (64.6)	44 (15.3)	6 (25.0)	16 (66.7)	2 (8.3)
**Southern Africa**	71 (10.8)	464 (70.6)	122 (18.6)	0 (0.0)	4 (80.0)	1 (20.0)
**Level of education**						
**Master’s degree and higher**	68 (10.2)	472 (70.7)	128 (19.2)	22 (26.8)	42 (51.2)	18 (22.0)
**Bachelor’s degree**	204 (16.5)	815 (65.9)	218 (17.6)	14 (23.0)	38 (62.3)	9 (14.8)
**Secondary/high school**	49 (11.2)	311 (71.3)	76 (17.4)	3 (33.3)	4 (44.4)	2 (22.2)
**Primary/no school**	19 (38.0)	29 (58.0)	2 (4.0)	0 (0.0)	1 (50.0)	1 (50.0)
**Occupation**						
**Non-healthcare**	157 (9.0)	1,188 (71.2)	313 (18.9)	17 (17.9)	61 (64.2)	17 (17.9)
**Healthcare**	183 (25.0)	439 (59.9)	111 (15.1)	22 (37.3)	24 (40.7)	13 (19.5)
**Working status**						
**Employed**	262 (15.1)	1,149 (66.3)	322 (18.5)	38 (27.3)	76 (54.7)	25 (18.0)
**Unemployed**	78 (11.9)	478 (72.6)	102 (15.5)	1 (6.7)	9 (60.0)	5 (33.3)
**Marital/family status**						
**Married**	126 (12.2)	719 (69.8)	185 (18.0)	29 (31.5)	47 (51.1)	16 (17.4)
**Not married ǂ**	214 (15.7)	908 (66.7)	239 (17.6)	10 (16.1)	38 (61.3)	14 (22.6)
**Religion**						
**Christians**	304 (14.2)	1,439 (67.2)	397 (18.6)	35 (25.4)	78 (56.5)	25 (18.1)
**Others**	36 (14.3)	188 (74.9)	27 (10.8)	4 (25.0)	7 (43.8)	5 (31.3)
**Smoking status**						
**Ex-smoker**	21 (14.8)	98 (69.0)	23 (16.2)	4 (22.2)	13 (72.2)	1 (5.6)
**Current smoker**	20 (11.9)	113 (67.3)	35 (20.8)	1 (12.5)	5 (62.5)	2 (25.0)
**Non-smoker**	299 (14.4)	1,627 (68.1)	424 (17.7)	34 (26.6)	67 (52.3)	27 (21.1)
**Have you been vaccinated for any condition**						
**No**	30 (7.0)	326 (75.8)	74 (17.2)	3 (20.0)	9 (60.0)	3 (20.0)
**Yes**	310 (14.2)	1,301 (66.3)	350 (17.9)	36 (25.9)	76 (54.7)	27 (19.4)
**Any pre-existing conditions**^**§**^						
**No**	280 (13.9)	1,383 (68.4)	359 (17.6)	23 (21.5)	61 (57.0)	23 (21.5)
**Yes**	60 (16.3)	244 (66.1)	65 (17.6)	16 (34.0)	24 (51.1)	7 (14.9)
Knowledge^*^	18.7 ± 4.9	18.5 ± 6.3	19.6 ± 3.6	22.7 ± 3.4	18.9 ± 6.0	20.5 ± 3.8
Attitude^*^	1.2 ± 2.2	0.9 ± 2.1	1.0 ± 2.0	0.7 ± 1.9	0.7 ± 1.8	1.3 ± 2.2
Perception^*^	6.7 ± 2.7	5.6 ± 3.1	5.8 ± 2.3	7.2 ± 2.4	5.3 ± 3.1	5.9 ± 2.9

^Data presented in frequencies (percentages)^

^*Data presented as mean ± standard deviation^

ǂ includes single, divorced and widowed.

^**§**includes the presence of any of the following conditions: cancer, diabetes, hypertension, asthma, kidney disease, any heart condition, sickle cell anemia^

COVID-19 vaccine uptake was highest among Central African residents (20.1%) who lived locally but was highest among West Africans (29.9%) in the diaspora. The uptake of COVID-19 vaccine was highest among those with primary education or less (38.0%) while among those in the diaspora, uptake was highest among respondents with a master’s degree or higher (26.8%). Resistance was substantial in those with Master’s and higher degree respondents (70.7% for locals and 51.2% for those in the diaspora). For both healthcare and non-healthcare workers in both groups, the greatest proportions were resistant to taking the vaccine. The proportion of uptake, hesitancy and resistance towards COVID-19 vaccines varied with the employment status of the respondents, though the unemployed had the highest proportions of vaccine resistance in both groups (72.6% for locals and 60.0% for the diaspora). Christians represented the higher number of those who said they were hesitant to take the vaccine (18.6%) as compared with non-Christians in the diaspora (31.3%). Those who were ex-smokers had the highest proportion of those who were resistant among both the locals (69.0%) and those in the diaspora (72.2%). The uptake of the vaccine was also higher among those with pre-existing conditions in both local and diasporan respondents.

Higher mean scores for attitude and perception were observed among the COVID-19 vaccine uptake respondents for the local residents, while the mean knowledge score was highest for the hesitant group. Among the diasporans, the mean knowledge and perception scores were similarly highest in uptake respondents, but a higher score for attitude was observed in the hesitancy respondents (Table 2).

### Unadjusted analysis of factors associated with COVID-19 vaccine uptake, resistance and hesitancy in SSA

Table 3 shows the unadjusted relative risk of factors associated with resistance and hesitancy towards COVID-19 vaccination among SSA respondents living locally and in the diaspora. Among the local residents, female sex was associated with the COVID-19 vaccine resistance [RR = 0.73, 95% CI: 0.58 – 0.93]. Local residency in East and Southern Africa was significantly associated with COVID-19 vaccine resistance [RR = 3.05, 95% CI: 1.31—7.08 and RR = 1.49, 95% CI: 1.12—2.00 respectively] and hesitancy [RR = 4.50, 95% CI: 1.83—11.02 and RR = 1.54, 95% CI: 1.09—2.19 respectively]. Having primary or less education was also associated with a lower risk of COVID-19 vaccine resistance [RR = 0.22, 95% CI: 0.12 – 0.41] and hesitancy [RR = 0.06, 95% CI: 0.01 – 0.25] among local residents.

**Table 3:** Relative risk (RR) for factors associated with COVID-19 vaccine uptake, hesitancy and resistance among SSA locals and diasporans. The base reference was COVID-19 vaccine uptake for all variables

Variable	LOCAL		DIASPORA	
	**Resistant RR (95%CI)**	**Hesitant RR (95%CI)**	**Resistant RR (95%CI)**	**Hesitant RR (95%CI)**
**Age in years**				
**18 – 28**	1.00	1.00	1.00	1.00
**29 – 38**	0.88 (0.66—1.18)	1.18 (0.83—1.67)	0.98 (0.15—6.50)	2.17 (0.26—17.89)
**39 – 48**	1.06 (0.76—1.47)	1.03 (0.68—1.55)	0.13 (0.03—0.65)	0.22 (0.03—1.47)
**49 + **	1.27 (0.84—1.92)	1.51 (0.93—2.46)	0.17 (0.03—0.84)	0.16 (0.02—1.12)
**Sex**				
**Males**	1.00	1.00	1.00	1.00
**Females**	0.73 (0.58—0.93)	0.83 (0.62—1.10)	2.13 (0.83—5.43)	1.66 (0.53—5.25)
**Region**				
**West Africa**	1.00	1.00	1.00	1.00
**East Africa**	3.05 (1.31—7.08)	4.50 (1.83—11.02)	-	-
**Central Africa**	0.73 (0.53—1.02)	0.68 (0.44—1.05)	1.58 (0.56—4.45)	0.51 (0.09—2.76)
**Southern Africa**	1.49 (1.12—2.00)	1.54 (1.09—2.19)	-	-
**Level of education**				
**Master’s degree and more**	1.00	1.00	1.00	1.00
**Bachelor’s degree**	0.58 (0.43—0.77)	0.57 (0.40—0.81)	1.42 (0.64—3.17)	0.79 (0.28—2.23)
**Secondary/High School**	0.91 (0.62—1.36)	0.82 (0.52—1.31)	0.70 (0.14—3.40)	0.81 (0.12—5.42)
**Primary/Less**	0.22 (0.12—0.41)	0.06 (0.01—0.25)	-	-
**Occupation**				
**Non-healthcare**	1.00	1.00	1.00	1.00
**Healthcare**	0.32 (0.25—0.40)	0.30 (0.22—0.41)	0.30 (0.14—0.67)	0.59 (0.23—1.54)
**Working status**				
**Employed**	1.00	1.00	1.00	1.00
**Unemployed**	1.40 (1.06—1.84)	1.06 (0.76—1.49)	4.0 (0.55—36.83)	7.60 (0.84—68.97)
**Marital/family status**				
**Married**	1.00	1.00	1.00	1.00
**Not married **^**ǂ**^	0.74 (0.58—0.95)	0.76 (0.57—1.02)	2.34 (1.02—5.41)	2.54 (0.92—7.00)
**Religion**				
**Christians**	1.00	1.00	1.00	1.00
**Others**	1.10 (0.76—1.61)	0.57 (0.34—0.97)	0.79 (0.22—2.86)	1.75 (0.43—7.18)
**Smoking status**				
**Ex-smoker**	1.00	1.00	1.00	1.00
**Current smoker**	1.21 (0.62—2.36)	1.60 (0.71—3.58)	1.54 (0.14—17.33)	8.0 (0.31—206.37)
**Non-smoker**	1.01 (0.62—1.65)	1.12 (0.61—2.06)	0.61 (0.18—2.00)	3.18 (0.34—30.10)
**Have you been vaccinated for any condition**				
**No**	1.00	1.00	1.00	1.00
**Yes**	0.39 (0.26—0.57)	0.46 (0.29—0.72)	0.70 (0.18—2.76)	0.75 (0.14—4.01)
**Any pre-existing conditions**^**§**^				
**No**	1.00	1.00	1.00	1.00
**Yes**	0.82 (0.60—1.12)	0.84 (0.58—1.24)	0.57 (0.26—1.25)	0.44 (0.15—1.26)
**Knowledge**	0.99 (0.97—1.01)	1.03 (1.01—1.06)	0.82 (0.73—0.91)	0.87 (0.77—0.98)
**Attitude**	0.96 (0.91- 1.01)	0.96 (0.90 – 1.03)	0.99 (0.80 – 1.24)	1.15 (0.90 -1.46)
**Perception**	0.88 (0.84—0.92)	0.90 (0.85—0.94)	0.77 (0.66 – 0.90)	0.84 (0.70 – 1.01)

If 95% confidence intervals (CI) around RRs that lies between 1 00 indicate not statistically significant. All comparisons were made against vaccinated pregnant women (RR=1.0)

^ǂ^includes single, divorced, and widowed

^§^includes the presence of any of the following conditions: cancer, diabetes, hypertension, asthma, kidney disease, any heart condition, sickle cell anemia

Unemployment was significantly associated with higher risk of vaccine resistance [RR = 1.40, 95% CI: 1.06 – 1.84] among local residents. Being unmarried [RR = 0.74, 95% CI: 0.58 – 0.95], and having a history of vaccination for other conditions were associated with lower risk of vaccine resistance [RR = 0.39, 95% CI: 0.26 – 0.57] among locals. Also, those with high risk perception scores were significantly less likely to resist [RR = 0.88, 95% CI: 0.84—0.92] or be hesitant [RR = 0.90, 95% CI: 0.85—0.94] to the COVID-19 vaccines.

For those in diaspora, older age (> 38 years) [RR = 0.13, 95% CI: 0.03—0.65], working in healthcare sector [RR = 0.32, 95% CI: 0.25 – 0.40], having a more knowledge [0.82, 95% CI: 0.73—0.91] and better perception scores [RR = 0.77, 95% CI: 0.66 – 0.90], were associated with lower risk of COVID-19 vaccine resistance. Those who were not married were more likely to resist the COVID-19 vaccines compared with the married persons [RR = 2.34, 95% CI: 1.02—5.41].

### Adjusted analysis of factors associated with COVID-19 vaccine uptake, resistance and hesitancy in SSA

Table 4 presents the associated factors of COVID-19 vaccine resistance and hesitancy in this study. After controlling for potential confounders in the local resident group, East African respondents were more likely to be resistant [ARR = 3.33, 95% CI: 1.40—7.94] and hesitant [ARR = 4.64, 95% CI: 1.84—11.70] towards receiving COVID-19 vaccines while Central African respondents were less likely to be resistant [ARR = 0.46, 95% CI: 0.32—0.68] or hesitant [ARR = 0.44, 95%CI: 0.27—0.72] towards the vaccines. Having a bachelor’s degree [ARR = 0.54, 95% CI: 0.38—0.76] or lower, being a health care worker [ARR = 0.24, 95% CI: 0.18—0.32], being previously vaccinated for any condition [ARR = 0.45, 95% CI: 0.30—0.69], and having a lower risk perception score [ARR = 0.86, 95% CI: 0.82 – 0.90] were associated with reduced risk of being resistant towards the COVID-19 vaccines among local residents in SSA. Among those in the diaspora, respondents who were aged 49 years and older [ARR = 0.17, 95% CI: 0.03 – 0.95], healthcare sector workers [ARR = 0.25, 95% CI: 0.10—0.62], as well as those with lower knowledge scores [ARR = 0.82, 95% CI: 0.73 – 0.91] were less likely to resist taking the COVID-19 vaccines.

**Table 4:** Adjusted relative risk (ARR) for factors associated with vaccine hesitancy among SSA residents living in (Locals) and outside of Africa (Diaspora). The base reference was COVID-19 vaccine uptake for all variables

Variable	Local		Diaspora	
	**Resistant**	**Hesitant**	**Resistant**	**Hesitant**
	**ARR (95%CI)**	**ARR (95%CI)**	**ARR (95%CI)**	**ARR (95% CI)**
**18 – 28 years**	1.00	1.00	1.00	1.00
**29 – 38 years**	1.32 (0.94—1.85)	1.89 (1.26—2.84)	1.35 (0.18 – 10.07)	2.6 (0.30 – 23.17)
**39 – 48 years**	1.28 (0.867—1.89)	1.29 (0.80—2.07)	0.21 (0.04 – 1.17)	0.29 (0.04 – 2.08)
**49 + years**	1.39 (0.86—2.23)	1.74 (1.00—3.05)	0.17 (0.03 – 0.95)	0.15 (0.02 – 1.16)
**West Africa**	1.00	1.00	-	-
**East Africa**	3.33 (1.40—7.94)	4.64 (1.84—11.70)	-	-
**Central Africa**	0.46 (0.32—0.68)	0.44 (0.27—0.72)	-	-
**Southern Africa**	1.32 (0.94—1.84)	1.39 (0.94—2.06)	-	-
**Master’s & above**	1.00	1.00	-	-
**Bachelor’s degree**	0.54 (0.38—0.76)	0.60 (0.40—0.90)	-	-
**Secondary/ high School**	0.52 (0.31—0.87)	0.56 (0.31—1.02)	-	-
**Primary & Less**	0.15 (0.07—0.32)	0.05 (0.01—0.24)	-	-
**Non-health care**	1.00	1.00	1.00	1.00
**Health care**	0.24 (0.18—0.32)	0.19 (0.13—0.27)	0.25 (0.10—0.62)	0.46 (0.16—1.35)
**Christians**	1.00	1.00	-	-
**Others**	0.96 (0.64—1.46)	0.50 (0.29—0.86)	-	-
**Vaccinated for any condition**	0.45 (0.30—0.69)	0.48 (0.29—0.77)	-	-
**Knowledge**	1.02 (1.00 – 1.05)	1.07 (1.04 – 1.11)	0.82 (0.73 – 0.91)	0.88 (0.77 – 0.99)
**Perception**	0.86 (0.82 – 0.90)	0.85 (0.80 – 0.90)	-	-

If 95% confidence intervals (CI) around RRs that lies between 1.00 indicate not statistically significant. All comparisons were made against vaccinated pregnant women (RR = 1.0)

Regarding COVID-19 vaccine hesitancy among local residents in SSA, the significant factors included East and Central African origin, aged between 29 – 38 years, being a health care worker, having a bachelor’s degree or less, non-Christians, having been previously vaccinated for other conditions, higher knowledge and lower perception scores. For those in the diaspora, being a healthcare worker [ARR = 0.46, 95% CI: 0.16—1.35] and having lower knowledge scores [ARR = 0.88, 95% CI: 0.77 – 0.99] were the factors that were significant for being hesitant.

## Discussion

The purpose of this study was to compare the uptake, resistance and hesitancy of the COVID-19 vaccine between locally resident SSAs and those in the diaspora. Uptake of the COVID-19 vaccine was found to be twice as high among residents in the diaspora compared to local SSA residents. The WHO and Centers for Disease Control and Prevention (CDC) have suggested that the low vaccination rates in low-and-middle-income countries is in part, due to inequitable distribution of vaccines. Accessibility to vaccines may have played a role in the low uptake rates in our study. Half of the 52 African countries that had received vaccines had only vaccinated up to 2% of their population at the time of this study, and 15 countries had vaccinated up to 10% [41]. However, majority of those residing in Africa and the diaspora were either resistant or hesitant to get vaccinated. This finding is different from that reported in a previous study [42] where a higher proportion of African residents and those in the diaspora were willing to accept the vaccine when offered. A survey conducted by CDC Africa prior to the introduction of vaccines on the continent found that the willingness to take the vaccine in 15 African countries ranged from 59 to 93% [43], which was in contrast with our findings of greater resistance towards COVID-19 vaccination. Studies conducted in the US and UK showed that Africans/Blacks were 13 times more likely to be hesitant than Whites [44, 45] which is similar to the high proportions of SSA in diaspora who were either hesitant or resistant to taking COVID-19 vaccines.

Socio-demographic characteristics have been shown to play significant roles in vaccine hesitancy and resistance [45]. In this study, age, region of origin, educational level, occupation and religion were significantly associated with either vaccine hesitancy or resistance among local and diasporan residents. Younger age groups among the local residents were almost twice likely to be hesitant and older age groups were less likely to be resistant to vaccines. This finding is consistent with other previous studies [32, 37, 45, 46], and may also be related to the fact that COVID-19 is more likely to present in the severe form among older age groups, making them more likely to accept the vaccine for their protection.

Local East African respondents were three times more likely to resist and almost five times more likely to be hesitant than West Africans. This may be due to misinformation about COVID-19 [47] and its vaccines [48] which was reported to be more common in East African countries such as Tanzania. The results showed that the least educated respondents were less likely to be resistant or hesitant. This may be as a result of not comprehending the scientific arguments being advanced against the vaccines and having to make choices based on past experiences or the information they do understand. A recent study in the US showed a similar pattern with those with lower levels of education showing less hesitancy than those with higher [48]. This is contrary to the results obtained in other studies [35–37, 41, 42]. A statement by a 61 year old on Africa news may provide an insight into the mindset of those who are less educated thereby making them more likely to accept vaccination: "If in the time of our mothers, in the time we were little children if these "WhatsApp doctors" had existed (people who post unreliable medical information on social media) I think we would have all died because our mothers who did not go to school agreed to vaccinate us against smallpox, measles, polio – all the other diseases without debate. Today, we are more educated, but curiously, we refuse vaccination. This is a certain danger for our society, according to what I have read here and there. The Congo is being blacklisted because we risk many deaths if we don't accept vaccination” [49].

Both local and diasporan healthcare workers showed less likelihood of being either resistant or hesitant as compared to non-healthcare workers in this study. Resistance and hesitancy have been found among health workers though lower when compared to non-healthcare workers [50–55]. However, Blacks /African health workers still show higher risk than their counterparts of being resistant/hesitant irrespective of the country they are in. Vaccine resistance and/or hesitancy is a hindrance to the vaccination campaign, as such, health workers who should be well educated about the vaccines are likely to exert an influence on others and possibly deter them from getting vaccinated. Most findings in the cited papers found that the fear of side effects was usually the reason for hesitancy and resistance among health workers. [51–53]

Among the local residents, individuals from other religions were less likely to be vaccine hesitant compared to those of the Christian faith. Religion has been reported to play a huge role in the life of Africans and influences their health seeking behavior [56, 57]. Olagoke et al. reported that some religious views have contributed to the rejection of vaccination [58]. However, an intervention study conducted among American Christians [59], showed that with proper presentation of scientific facts, such negative views can be changed. Community engagement with religious leaders has also been advocated as a means of addressing vaccine hesitancy [60].

Local residents who had been previously vaccinated for other conditions were less likely to be COVID-19 vaccine resistant or hesitant. This finding emphasizes the influence of past experiences which can build confidence in the efficacy of vaccines. Other studies have also shown a willingness to be vaccinated among those who had previously received vaccinations for other diseases such as flu, yellow fever, hepatitis [61, 62]. Knowledge of COVID-19 vaccine was a significant factor among both local and diasporan residents. Knowledge has been shown to reduce resistance to vaccine acceptance. Africans in the diaspora were less likely to be hesitant or resistant to vaccines as compared to their counterparts residing in Africa. This may still be related to misinformation and the need for health messages to be relayed in the languages familiar to the people. Recent studies have shown a decline in those who are hesitant and this has been attributed to the availability of accurate information that reduces fear and leads to making informed decisions [63]. Exposure to accurate information and increased knowledge about COVID-19 vaccines may help those who are hesitant to be more receptive to vaccines. Among local residents, higher perception scores showed a lower odd of being either resistant or hesitant. The perception that one is likely to be at risk of contracting a disease can result in people taking appropriate measures to protect themselves from contracting the disease.

### Strengths and limitations

This is the first large scale study to compare acceptance of COVID-19 vaccines between sub-Saharan African local residents and those in the diaspora. The study employed robust analyses to control for potential confounders to reduce the possibility of a bias. The distribution of the questionnaire in both English and French languages was through an internet-based methodology, which was the only reliable means to disseminate information at the time of this study to a wider audience. Notwithstanding these strengths, the study has some limitations. For example, the study did not explore concerns about vaccine safety which may be an important determinant of vaccine hesitancy. The cross-sectional nature of the study means that causation cannot be determined. The survey was distributed electronically using social media platforms and emails, and this may have inadvertently excluded some potential participants whose opinions may have differed, such as those without internet access and people living in rural areas, where internet penetration remains relatively low [64]. The survey was presented in English and French and thus inadvertently excluding some of the Portuguese or Arabic-speaking SSA countries from participating. Although the study showed satisfactory internal validity, its generalization or transferability to all SSA countries may be limited. Despite the wide distribution of the survey, only few SSA living in diaspora participated compared to many who lived in SSA. However, robust analysis was conducted through the use of proportions for comparison and the use of regression analysis to ensure adequate control of potential confounders.

## Conclusions

The study showed that Africans residing both locally and in diaspora are mostly either resistant or hesitant to the COVID-19 vaccines. Factors that influenced resistance and hesitancy among local residents included younger age, East and Central African residency, lower levels of education, history of previous vaccinations, being a healthcare sector worker, knowledge and perceptions of COVID-19 vaccine. For Africans in the diaspora, being hesitant or resistant to COVID-19 vaccines are influenced by older age, being a healthcare sector worker and having adequate knowledge of vaccines. The implication of these findings is that continued hesitancy and resitance to the COVID-19 vaccines by Africans irrespective of where they are based could jeopardise the progress made by countries in the distribution of the vaccines and subsequent control of the spread of the virus. Future research could explore if the factors identified in this study are also playing a role in hesitancy/resitance to other vaccines. Appropriate interventions such as public health messaging that take into consideration the different influencing factors are required to enhance COVID vaccine uptake to achieve sufficient vaccine coverage.

## Supplementary Information

**Additional file 1:**

## Data Availability

The datasets used and/or analysed in this study are available from the corresponding author on reasonable request.

## Abbreviations

ARR: adjusted relative risk
CDC: Centers for Disease Control and Prevention
CI: confidendence interval
COVID-19: Coronavirus disease 2019
RR: relative risk
SSA: sub-Saharan Africa
UK: United Kingdom
USA: United States of America
WHO: World Health Organization
