# Supplementary material for: Acceptance of COVID-19 vaccine among sub-Saharan Africans (SSA): a comparative study of residents and diasporan dwellers
Source: BMC Public Health. 2023 Jan 28;23:191. doi: 10.1186/s12889-023-15116-w (PMC9884132; doi:10.1186/s12889-023-15116-w)
Supplement: Supplementary file 1 — Additional file 1: [file 12889_2023_15116_MOESM1_ESM.docx]

Supplementary file S1 Table

| Demography |
| --- |
| 1. **Country of Origin** |
| - West Africa |
| - East Africa |
| - Central Africa |
| - Southern Africa |
| 1. **Country of residence** |
| - Local |
| - Diaspora |
| 1. **Age categories in years** |
| - 18-28 years |
| - 29-38 |
| - 39-48 |
| - 49+years |
| 1. **Gender** |
| - Males |
| - Females |
| 1. **Marital Status** |
| - Married/Defacto |
| - Single |
| - Separated/divorced |
| - widowed |
| 1. **Highest level of Education** |
| - Postgraduate Degree (Masters /PhD) |
| - University degree |
| - Diploma |
| - Secondary/High School |
| - Primary Education |
| - No formal education |
| 1. **Employment status** |
| - Employed |
| - Self employed |
| - Unemployed/Retired |
| 1. **Religion** |
| - Christianity |
| - Islam |
| - African Traditional |
| 1. **Occupation** |
| - Student |
| - Healthcare worker/professional |
| - Non healthcare worker |
| - Others |
| 1. **Smoking status (Yes/No)** |
| - Ex-smoker |
| - Current smoker |
| - Non-smoker |
| 1. **Pre-existing Health conditions (Yes/No)** |
| - Cancer |
| - Kidney Disease |
| - Any Heart condition |
| - Diabetes |
| - Sickle cell anaemia |
| - Hypertension |
| - Asthma |
| 1. Have you ever been vaccinated for any condition before? Yes/No/Can't remember |
| 1. Which condition(s) where you vaccinated against? (You can select more than one option) |
| - Tuberclosis |
| - MMR |
| - Yellow Fever |
| - Polio |
| - DPTT |
| - Flu |
| - Whooping Cough |
| - BCG vaccine |
| - Chickenpox (Varicella) |
| - Hepatitis |
| **General knowledge of vaccination**   1. Have you heard about community (herd) immunity? [Yes/No] |
| 1. Immunity from a disease can occur through the following [Strongly agree/agree/not sure/disagree/strongly disagree] |
| - Natural immunity |
| - Acquired from personal infection and recovery |
| - Through vaccination |
| - No one has immunity from any disease |
| 1. You can get a disease after you have been vaccinated against that disease [Strongly agree/agree/not sure/disagree/strongly disagree] |
| 1. Having been vaccinated against one disease protects me from other disease [Strongly agree/agree/not sure/disagree/strongly disagree] |
| 1. Having been vaccinated against a disease can reduce the severity of the disease [Strongly agree/agree/not sure/disagree/strongly disagree] |
| 1. Are you aware that vaccines have been developed to protect people against COVID-19? [Yes/No] 2. COVID-19 vaccine can prevent covid-19 infection and its complications [Strongly agree/agree/not sure/disagree/strongly disagree] |
| **Perception of COVID-19** |
| Please rate your chances of personal risk of infection with COVID-19 for each of the following?   1. your risk of becoming infected [Very high/high/unlikely/low/very low] |
| 1. your risk of dying from the infection [Very high/high/unlikely/low/very low] |
| **COVID-19 Vaccination** |
| 1. Have you been vaccinated against COVID-19? [Yes/No] |
| 1. Will you be willing to be vaccinated against COVID-19 if the vaccine becomes available in your country [Yes/No/Not sure] |
| **Attitude** |
| 1. Does taking a COVID-19 vaccine mean that you should stop other measures [Yes/No/Not sure] |
| - Facemask |
| - Practicing Physical/Social distancing |
| - Using alcohol-based hand sanitizers |
| - having to avoid large gatherings/crowded places |
